# Photoelectric properties of glass-ceramics containing KTb_2_F_7_ nanocrystals for UV detection†

**DOI:** 10.1039/d3ra05044k

**Published:** 2023-10-10

**Authors:** Zhiguo Zhao, Xian Zhang, Xuying Niu, Rui Zhang, Zaijin Fang, Zhi Chen, Hong Jia

**Affiliations:** a College of Physics and Electronic Information, Henan Key Laboratory of Electromagnetic Transformation and Detection, Luoyang Normal University Luoyang 471934 China jiahong517@aliyun.com zxian18237988270@163.com; b Department of Optoelectronics Science, Harbin Institute of Technology at Weihai Weihai 264209 China; c Guangdong Provincial Key Laboratory of Optical Fiber Sensing and Communications, Institute of Photonics Technology, Jinan University Guangzhou 511443 China zaijinfang@163.com; d Zhejiang Lab Hangzhou 311100 China; e Longmen Laboratory of Luoyang 471000 Luoyang 471934 China

## Abstract

In this work, a glass ceramics (GC) containing KTb_2_F_7_ nanocrystals was fabricated by controlled crystallization of an fluorosilicate glass *via* heat-treatment. The microstructure, luminescence, and photoelectric properties of the GCs are systematically studied by X-ray diffraction, transmission electron microscopy, spectral analysis, and current–voltage (*I*–*V*) curves. The results show that the GC containing KTb_2_F_7_ nanocrystals exhibit intense visible emission due to the 4f transition of Tb^3+^: ^5^D_*i*_ (*i* = 3, 4) → ^7^F_*j*_ (*j* = 0–6) upon excitation of ultraviolet (UV) light. In addition, a UV detector device based on the GC was fabricated, which has a large dynamic linear response range, fast response speed and high sensitivity. This study not only provides a new material for UV detector that can simplify the process of UV detection, but also highlight a new strategy for UV detection.

## Abstract 2

Oxyfluoride glass-ceramic containing KTb_2_F_7_ nanocrystals can convert UV light into visible light, and then the visible light is absorbed by Si-APD to produce a photocurrent. This UV detector has a large dynamic linear response range, fast response speed and high sensitivity.

## Graphical abstract

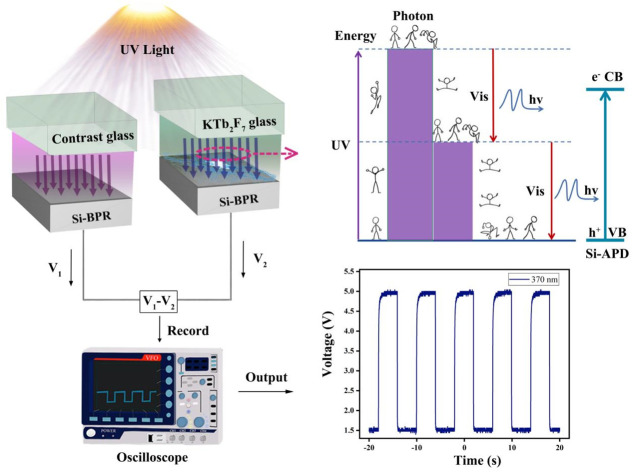

## 1. Introduction

Glass ceramics (GCs) have been widely investigated for applications in many fields, such as spectral conversion, information storage, optical communication and photoelectric detection, due to their excellent optical properties in ultraviolet (UV), visible and infrared regions.^1,2^ Owing to the high-efficiency and multi-wavelength luminescence, rare-earth (RE) ion doped GCs have been usually used as optical conversion materials to realize spectral modulation for photoelectric detection.^3–5^ Fluoride glasses are the preferred substrate for doping RE ions because of their low phonon energy that could suppress phonon-assisted nonradiative process. However, the application of fluoride glasses has been limited due to their susceptibility to corrosion and instability. In comparison, oxide glass is easy to be made into various shapes, exhibiting stable physical and chemical stability, and high mechanical strength. The oxyfluoride GCs in which fluoride crystals are dispersed in an oxide glass matrix bear the benefits of both oxide and fluoride glass, therefore they have been considered as ideal hosts or RE ions.^6–10^ In the past decades, RE-doped fluorosilicate GCs have been considered as the most commonly used luminescent materials because they feature a robust structure as well as low-phonon energy. In the fluorosilicate GCs, Si–O networks construct the frameworks of glasses and fluoride nanocrystals are precipitated from the glass network. The Si–O frameworks are strong, and isolate the fluoride crystals from the outside environments, and increase the stability of glasses. The fluoride crystals provide low-phonon energy coordination sites for RE ions that could enhance the luminescence efficiency.^11–21^ Thus, fluorosilicate GCs exhibit excellent thermal stability as well as high-efficiency luminescence. So far, a variety of fluorosilicate GCs have been designed for achieving high-efficiency luminescence.^22–29^ For example, Meyneng *et al.* proposed an optimized SiO_2_–Al_2_O_3_–YLiF_4_ system and obtained GCs containing YF_3_ or YLiF_4_ phase with controllable transparency through controlled heat treatment.^30^ By using the same melt-quenching method, Gorni *et al.* prepared transparent oxyfluoride GCs doped with Nd^3+^ and Er^3+^ ions. It is found that most of the RE ions are concentrated in the fluorine-rich amorphous regions in the parent glass, and the crystallization process triggers the redistribution and incorporation of RE ions in fluoride nanocrystals.^31^

Herein, a novel Tb^3+^ doped fluorosilicate glass is synthesized by melt-quenching method, and KTb_2_F_7_ nanocrystals are precipitated from the glass *via* the subsequent heat-treatment. The microstructure and optical properties of GC containing KTb_2_F_7_ nanocrystals are investigated by X-ray diffraction (XRD) patterns, transmission electron microscopy (TEM) images, transmission spectra, photoluminescence spectra, Raman spectra and FTIR spectra. The GC containing KTb_2_F_7_ nanocrystals exhibits strong visible emission under UV excitation. The GC is then employed as a spectral converter in a UV photodetector device, which demonstrates efficient photoelectric response to UV light. The present work suggests that the designed GCs materials are excellent candidates for solar blind detectors.^32–35^

## 2. Experimental

### 2.1 Sample synthesis

**Fig. 1:**
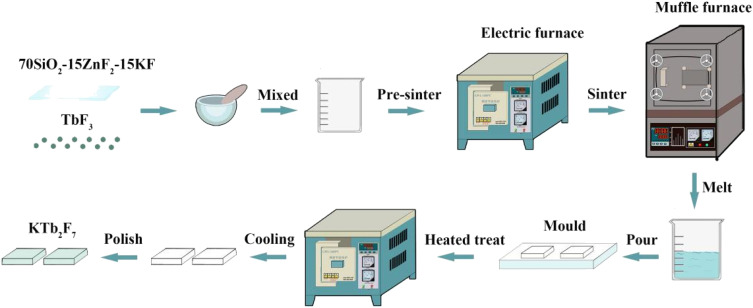
Process diagram of sample fabrication in this work.

The sample with a nominal composition of (mol%) 70SiO_2_–15KF–15ZnF_2_ is selected as the host glass. The glass is prepared by a melt-quenching method and TbF_3_ is used as the doping source. The doping concentration of Tb^3+^ ions in the glass is set to 0.15 mol%. The process of sample fabrication is present in Fig. 1. A stoichiometric mixture of 30 g of reagent grade SiO_2_ (99.99%), ZnF_2_ (99.99%), KF (99.99%) and TbF_3_ (99.99%) is mixed thoroughly and then melted in an alumina crucible at 1550 °C for 30 min. The crucible is covered during the glass melting process. The glass melt is poured onto a cold brass mold and pressed with another brass plate to prepare precursor glasses. Then, the precursor glasses are heat treated at 520 °C for 10 h to obtain the GCs.^32,36,37^

### 2.2 Characterizations

The crystal phase in NGCs was identified by X-ray diffractometer (Bruker, Flanden, Switzerland), and XRD pattern was performed by Cu/Ka (*λ* = 0.1541 nm) radiation. High-resolution transmission electron microscopy (HR-TEM) (Tecnai G2, FEI, USA) was used to characterize the morphology and size distribution of nanocrystals in GCs. The luminescence spectrum of the sample was recorded using the Edinburgh FLS980 fluorescence spectrometer (Edinburgh Instrument, Edinburgh, UK), and the transmission spectrum was measured by the ultraviolet/VIS/NIR spectrophotometer (Lambda-900, PerkinElmer, USA).

### 2.3 Design and measurement of the UV photodetection device

**Fig. 2:**
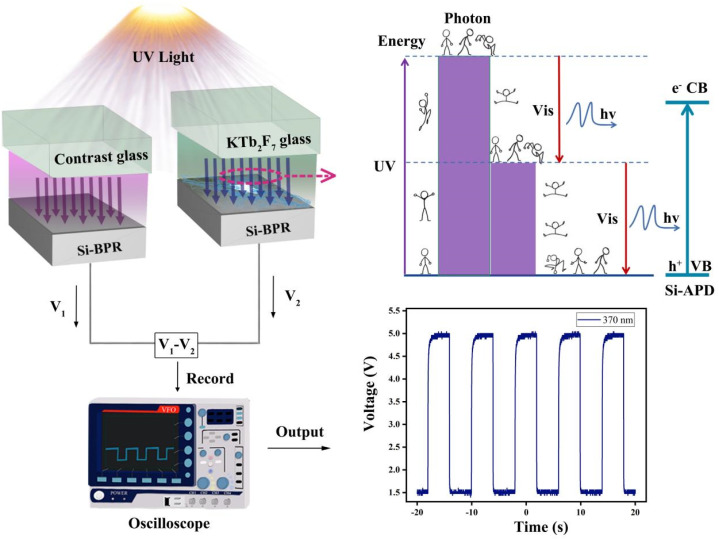
Mechanistic illustration of wide-band solar-blind UV detection based on KTb_2_F_7_ containing GCs.

The structure of the broadband UV detector designed in the present work is shown in Fig. 2, which consists of a silicon semiconductor (Si-APD) covered by a spectral converter. Here, the fabricated KTb_2_F_7_ containing GC is employed as the spectral converter, which is directly excited by external UV light source. These UV-excited visible emission from the GC is then absorbed by a silicon semiconductor (Si-APD) and a subtraction design is used to detect the optical voltage response. The photoelectric measurement is performed under irradiated by UV light, and the GC containing KTb_2_F_7_ nanocrystals can effectively convert the UV light into visible light. The photovoltage of the silicon photoresistor covered with the GCs containing KTb_2_F_7_ nanocrystals under UV irradiation is calculated according to the difference between the silicon photoresistor covered with the GCs and the control glass under UV irradiation. The photovoltage of the silicon photoresistor covered with the convertor GC under pulse UV excitation is recorded by an oscilloscope.^29,38,39^

## 3. Results and discussion

**Fig. 3:**
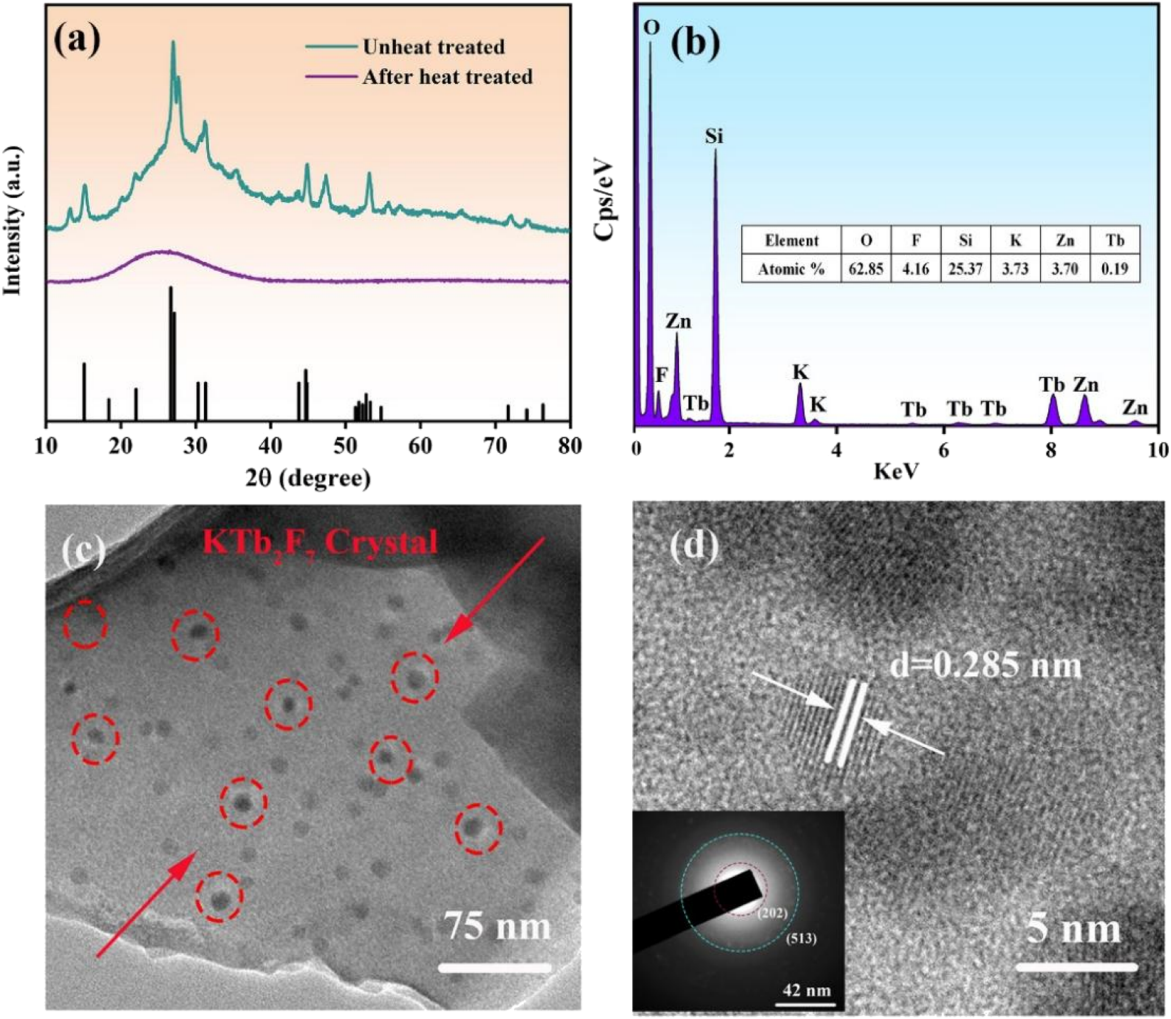
(a) XRD patterns of the parent glass and the GC obtained after heat-treatment; (b) EDS mapping for the KTb_2_F_7_ containing GC; (c) TEM image; (d) HR-TEM image and SAED image.

In order to determine the crystalline phase, XRD patterns of glass and GC samples are measured, as shown in Fig. 3a. A broad hump is observed in the XRD pattern of precursor glass due to the amorphous nature, indicating that no crystals are precipitated in the precursor glass. However, sharp peaks are observed in the XRD patterns of the GC, which match well with those of the standard card of KTb_2_F_7_ crystals (PDF#32-0849). The peaks at 18.277, 31.25, 43.737, 51.751, 76.442 are attributed to (0 0 2), (2 0 2), (3 0 0), (−3 1 3), (5 1 3) crystal facets of KTb_2_F_7_, respectively. These observations prove that only KTb_2_F_7_ crystal is precipitated in the GC after heat-treatment. The GC sample was further examined by using energy dispersive X-ray spectroscopy. As shown in Fig. 1b, the obtained elemental composition agrees with the nominal ratio of raw materials used to fabricate the parent glass.

To study the microstructure of samples, TEM and HR-TEM images of the GC samples are measured, as shown in Fig. 3c and d. It is found that the nanoparticles are uniformly dispersed in the glass matrix with a mean diameter from 10 to 50 nm (Fig. 3c). The HR-TEM image in Fig. 1d shows that the crystal lattice fringes can be clearly observed. The inter-plannar distances in the crystal lattice fringes are measured directly to 0.285 nm (Fig. 3c), which corresponds to the (2 0 2) crystal facet of KTb_2_F_7_ crystals. These results directly provide that KTb_2_F_7_ nanocrystals are precipitated in the GC. The selected area electron diffraction (SAED) pattern in Fig. 1d presents several diffraction rings that can be ascribed to the (2 0 2) and (5 1 3) crystal faces, confirming the polycrystalline diffraction characteristics of precipitated nanocrystals in the GC.

**Fig. 4:**
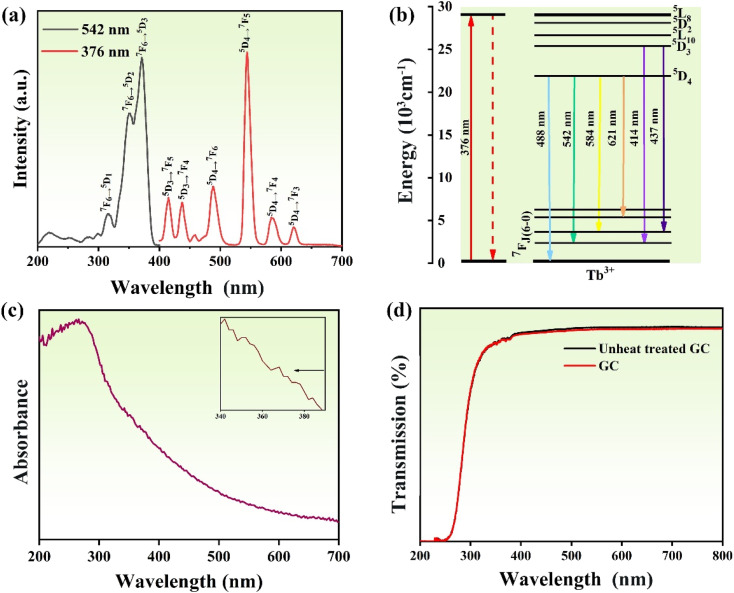
(a) Photoluminescence and excitation spectra of the KTb_2_F_7_ containing GC; (b) energy level diagram and the involved optical transitions of the Tb^3+^ ions in the GC; (c) absorption spectrum of KTb_2_F_7_ GC; (d) transmission spectrum of KTb_2_F_7_ containing GC.

Next, we characterize the optical properties of the sample glass by optical spectrum analysis. The excitation spectra of GC for the emission at 542 nm and photoluminescence spectra at excitation of 376 nm are shown in Fig. 4a. We can see that there are three main excitation peaks at 320 nm, 348 nm, and 376 nm, among which the peak at 376 nm is the typically strongest excitation peak. The corresponding ground transitions are ^7^F_6_–^5^D_1_, ^7^F_6_–^5^D_2_, and ^7^F_6_–^5^D_3_.^40^ In the photoluminescence spectrum from 400 nm to 700 nm, it is obvious that there are six luminescence peaks, which are located at 414 nm (^5^D_3_–^7^F_5_), 437 nm (^5^D_3_–^7^F_4_), 488 nm (^5^D_4_–^7^F_6_), 542 nm (^5^D_4_–^7^F_5_) and 584 nm (^5^D_4_–^7^F_4_) (Fig. 4b). Since the strongest emission peak is at 542 nm, the sample emits mainly yellow-green light under UV irradiation (as shown in the 5d illustration). Fig. 4c shows the absorption spectrum of the GCs. In the entire UV spectrum region, GC has strong and wide absorption bands. In addition, it can also be seen that there is weak absorption in the visible region, which agrees with the UV-visible transmission spectrum in Fig. 4d. Since the size of the precipitated KTb_2_F_7_ crystals is smaller than the wavelength of visible light and UV light, the glass-ceramics still have high transparency in the UV region. The FTIR spectrum of the GC is given in Fig. S1.† It can be seen that there are several sharp absorption peaks at 2878 cm^−1^, 2964 cm^−1^, 3410 cm^−1^, which correspond to the absorption by the 4f transitions of Tb^3+^ ions in KTb_2_F_7_ microcrystals, while the wide absorption peak near 2000 cm^−1^ could be ascribed to the absorption of precursor glass. In order to further measure the change of glass network structure, the Raman spectrum of the sample glass was analyzed, as shown in Fig. S2.† It can be seen that obvious Raman peaks appear near 796 and 1094 cm^−1^, which are Raman peaks caused by the glass matrix, while the sharp peak at 652 nm cm^−1^ is ascribed to KTb_2_F_7_ crystals, confirming again the precipitation of the fluoride phase in the GC, the Raman peak of GC, which causes the change of the structure of the silicon-oxygen tetrahedral unit and the change of Si–O vibration, thus causing the shift of the peak value.

**Fig. 5:**
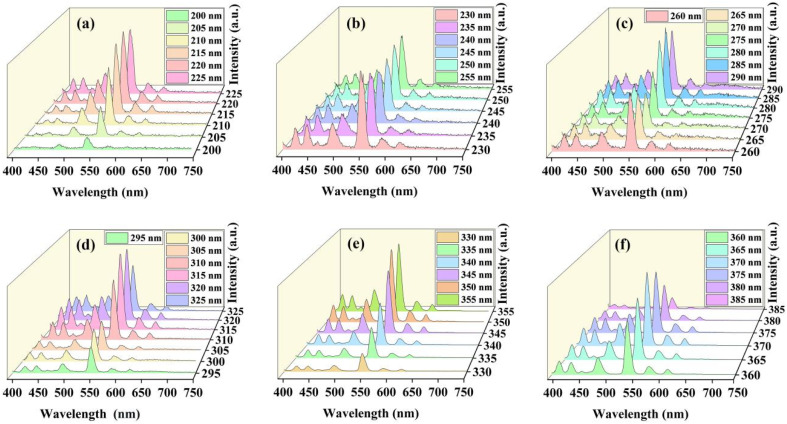
(a)–(d) Photoluminescence spectra of the KTb_2_F_7_ containing GC upon excitation at different wavelengths. (a) 200–225 nm; (b) 230–255 nm; (c) 265–290 nm; (d) 295–325 nm; (e) 330–355 nm; (f) 360–385 nm.

Next, the photoluminescence characteristics of the sample with the change of excitation wavelength has been demonstrated further. As shown in Fig. 5a–f, the emission intensity increases first and then decreases along with the excitation wavelength. For excitation wavelengths from 200 to 255 nm, the relative luminescence intensity always shows an increasing trend, and then it decreases significantly in the range of 230–245 nm, but it shows an increasing trend after 245 nm and then decreases significantly until the excitation wavelength reaches 260–270 nm. After that, the luminescence intensity increases rapidly between 275 and 315 nm, stabilized at 315–320 nm, and then decreased again. At the excitation wavelength longer than 330 nm, the emission intensity grows fast and the maximum emission intensity is observed for excitation wavelength of 370 nm. This result is consistent with the excitation spectrum shown in Fig. 4a, where an excitation peak is observed near 370 nm due to the ^7^F_6_–^5^D_3_. From the spectra presented in Fig. 5, the emission bands due to the set of ^5^D_*i*_–^7^F_*j*_ transitions exhibit a similar variation trend as the excitation wavelength changes from 200 nm to 385 nm.

**Fig. 6:**
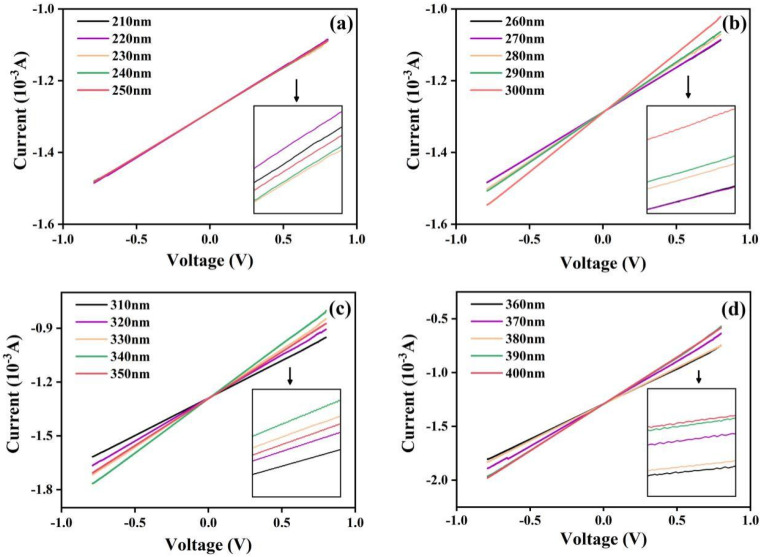
(a)–(d) *I*–*V* curves of the integrated UV photodetector based on the KTb_2_F_7_ containing GC upon excitation at different wavelengths. (a) 210–250 nm; (b) 260–300 nm; (c) 310–350 nm; (d) 360–400 nm.

To examine the photoelectric response, we recorded the *I*–*V* curves of the UV photodetector based on the KTb_2_F_7_ containing GC in the voltage range of −0.8 to 0.8 V under UV irradiation through an electrochemical workstation, and the results are shown in Fig. 6. With different excitation wavelengths, the photocurrents almost unchanged in the range of 210–270 nm, indicating that the current has almost no change with increase of wavelength, and that the photoelectric response is almost independent of the photon energy. For UV irradiation at 270 nm, it can be seen that the *I*–*V* curves are obviously separated, and the photocurrent increases first and then decreases. In the positive voltage range, the maximum value is reached when the wavelength increases to 340 nm. From 350 nm, the current value suddenly decreases, but after 350 nm, the current value increases slightly with the increase of wavelength. Since the strongest luminescence intensity of the GC is obtained under excitation at 370 nm, the highest current is observed for the device under 370 nm irradiation, and the current is much higher than that recorded under irradiation by UV at 380 nm (the same result can be obtained from the absolute value of negative voltage). These results on the photoelectric response of the device are consist with the above optical properties of KTb_2_F_7_ containing glass ceramics.

**Fig. 7:**
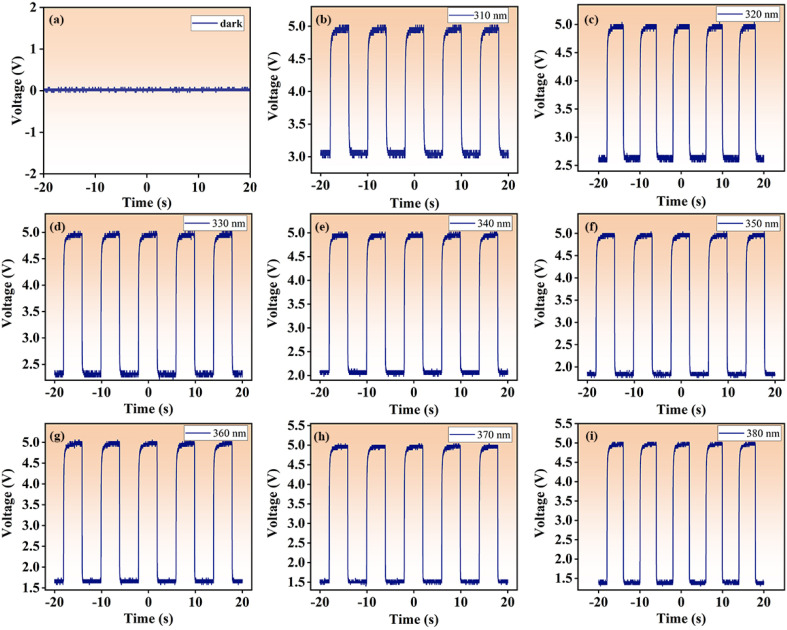
Photoelectric response of UV detector recorded under pulse UV light irradiation at different wavelengths. (a) Dark; (b) 310 nm; (c) 320 nm; (d) 330 nm; (e) 340 nm; (f) 350 nm; (g) 360 nm; (h) 370 nm; (i) 380 nm.

In order to further investigate the reproducibility and sensitivity of the UV detector, we built an optical switching device to try to capture the responding optical voltage trend through an oscilloscope, as plotted in Fig. 7. When the detector is placed in the dark, the voltage value is 0 V. Under UV light irradiation, positive voltage signal is observed, which proves that the optical signal from the GC can be effectively converted into electrical signal. It can also be observed that the overall oscilloscope pulse signal still approximately preserves the square waveform with little change in waveform with the change of UV wavelengths, suggesting the high reproducibility of the device. The photoelectric response curves are recorded for UV wavelengths with a 10 nm interval. By observing the voltage difference signals from 310 nm to 380 nm, the signal peaks all reaches saturation around 4 s, and the maximum signal value fluctuates the least at 370 nm, and the voltage is evenly distributed around 5 V. As is known, the visible luminescence intensity of the GC is the strongest at the excitation wavelength of 370 nm, this contribute to the shortest response time and the fastest voltage change of the device. The measured response time is about 0.8 s (see Fig. S3† in the attachment). The above observation also confirms that the noise is minimal at UV wavelength of 370 nm, validating that the characteristics of high sensitivity and high signal-to-noise ratio of our ultraviolet detector. Recently existing UV detector materials were investigated, as shown in Table 1. On the basis of this research, a new and more efficient UV detector is constructed by using GCs.

**Table 1:** Investigate the comparison of different solar blind UV detector materials^38^

Type	Material	Advantage	Disadvantage	Spectral window (nm)	Ref.
Wide bandgap semiconductors (WBGS)	Diamond/β-Ga_2_O_3_, AlGaN, CdMoO_4_–ZnO, r-GeO_2_, TiO_2_, SnO_2_*et al.*	Good thermal conductivity, high electron drift saturation speed, stable chemical properties *et al.*	Complex preparation process, high technical requirements *et al.*	214–350	41–43
				260–280	44
				300–390	45
				200–280	46
				200–290	46–49
Quantum dot (QD)	CsPbX_3_, MAPbCl_3_*et al.*	The absorption capacity of UV light is weak and so on	Most of the raw materials are toxic elements, existing security threat *et al.*	280–400	50
				340–400	51
DC luminophores	Gd_2_O_3_:Eu-PMMA, KTb_2_F_7_ GCs *et al.*	Broaden the corresponding range of the spectrum *et al.*	—	200–400	38
				300–400	This work

## 4. Conclusions

In conclusion, fluorine oxide glass ceramics (GCs) containing KTb_2_F_7_ nanocrystals were prepared by the melt-quenching process. The precipitation of crystalline KTb_2_F_7_ is confirmed XRD analysis, and the crystallization mechanism of the crystalline substance has been discussed. The TEM results further confirm that the cubic phase KTb_2_F_7_ nanocrystals are precipitated in the glass. The optical measurement results show that the broadband absorption characteristics of the fluoro-oxide glass ceramics in the ultraviolet range and strong visible luminescence characteristics of the GC. In the photoelectric measures, the developed GC shows strong response to UV light, which could be promising for application in UV defectors.

## Conflicts of interest

There are no conflicts to declare.

## Supplementary Material

RA-013-D3RA05044K-s001
