# Supplementary material for: Photoelectric properties of glass-ceramics containing KTb2F7 nanocrystals for UV detection
Source: RSC Adv. 2023 Oct 10;13(42):29419–26. doi: 10.1039/d3ra05044k (PMC10563034; doi:10.1039/d3ra05044k)
Supplement: RA-013-D3RA05044K-s001 [file RA-013-D3RA05044K-s001.pdf]

Electronic Supplementary Material (ESI) for RSC Advances.
This journal is © The Royal Society of Chemistry 2023

Supplement information

Photoelectric properties of glass-ceramics containing KTb_2F_7 nanocrystals for UV detection

Zhiguo Zhao[#a], Xian Zhang[#a, *b], Xuying Niu[a], Rui Zhang[a], Zaijin Fang[*c], Zhi Chen[d], and Hong Jia[*a, e]

[a]*College of Physics and Electronic Information & Henan Key Laboratory of Electromagnetic Transformation and Detection, Luoyang Normal University, Luoyang 471934, China.*

[b]*Department of Optoelectronics Science, Harbin Institute of Technology at Weihai, Weihai 264209, China.*

[c]*Guangdong Provincial Key Laboratory of Optical Fiber Sensing and Communications, Institute of Photonics Technology, Jinan University, Guangzhou 511443, China..*

[d]*Zhejiang Lab, Hangzhou 311100, China.*

[e]*Longmen Laboratory of Luoyang, 471000,Luoyang 471934, China*

E-mail: jiahong517@aliyun.com, zxian18237988270@163.com and zaijin_fang@163.com

# The authors contributed equally.

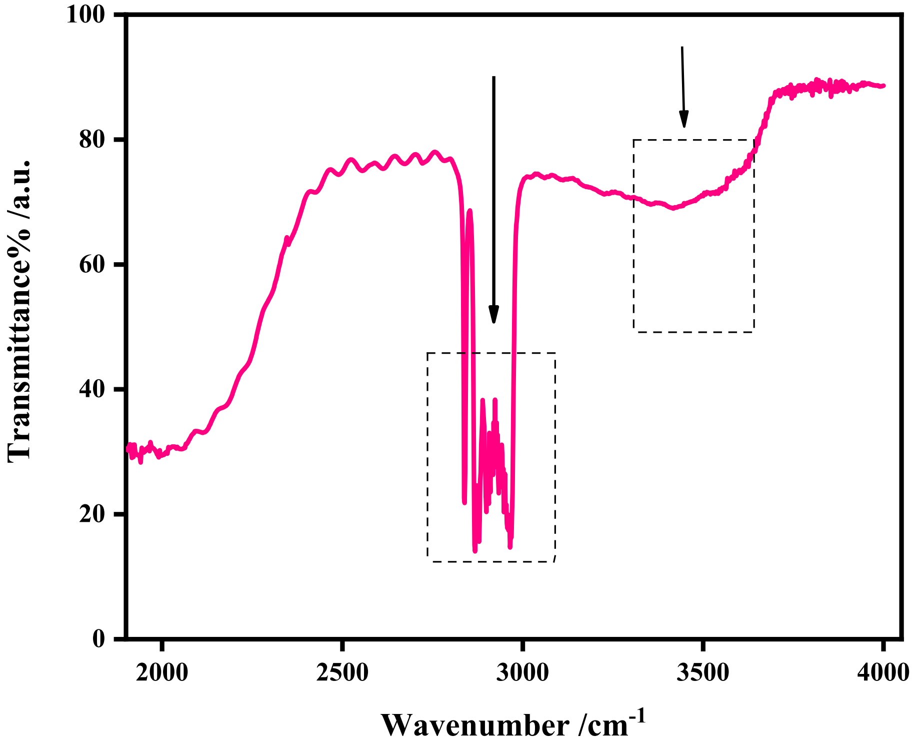

Fig. S1. FTIR spectrum of the glass-ceramics obtained after heat treatment.

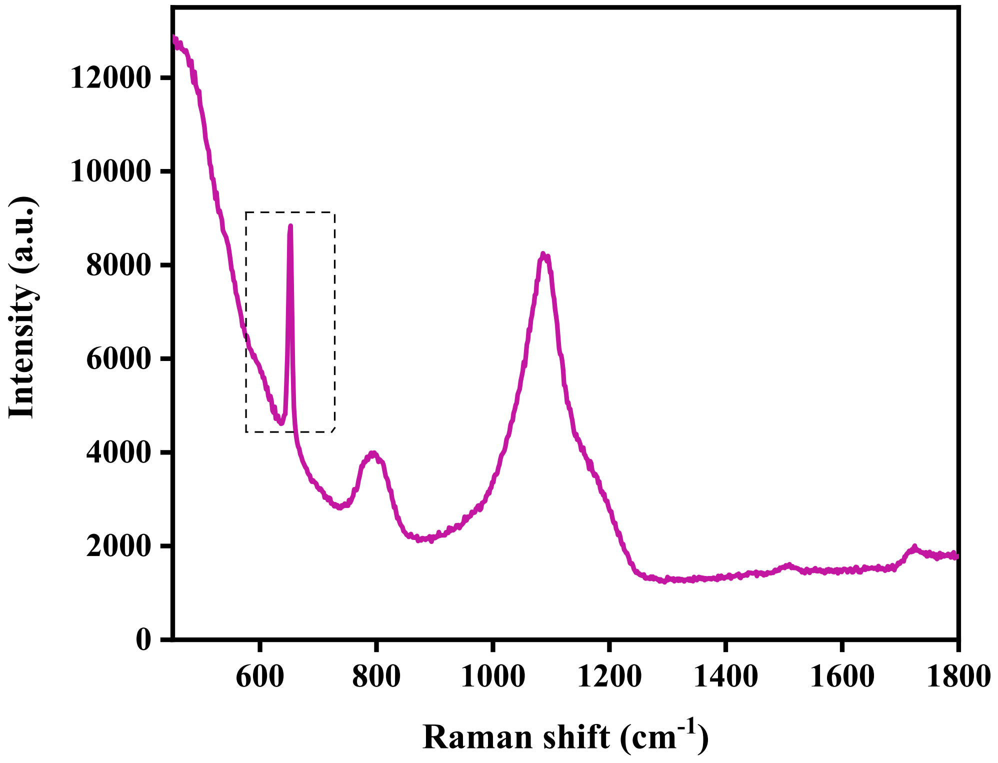

Fig. S1. Raman spectrum of the glass-ceramics obtained after heat treatment.

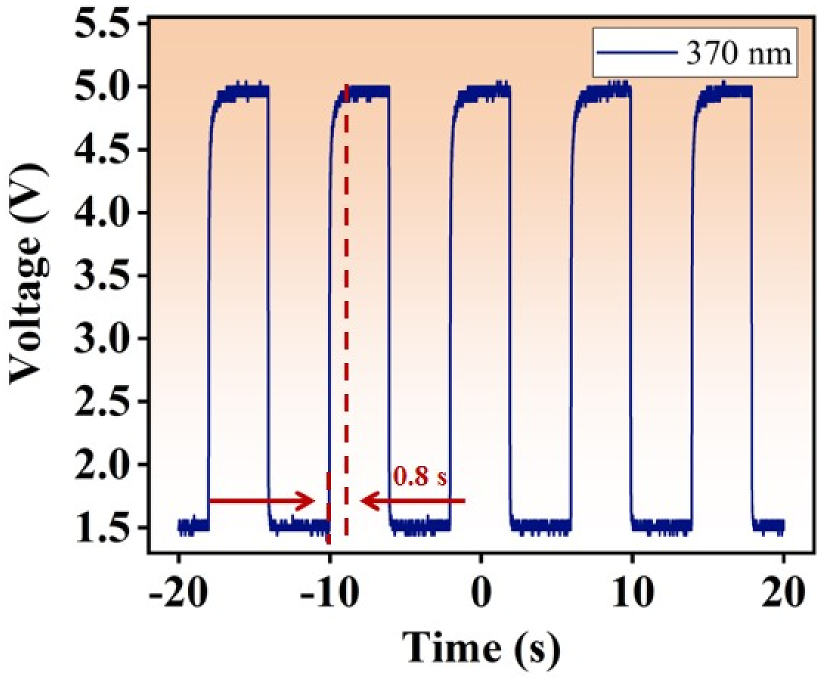

Fig. S3. Time-dependent photoresponse of the device based on the developed GC-based DC spectral converter under solar-blind UV light irradiation (370 nm).
